# Natriuretic peptides system in the pulmonary tissue of rats with heart failure: potential involvement in lung edema and inflammation

**DOI:** 10.18632/oncotarget.24922

**Published:** 2018-04-24

**Authors:** Emad E. Khoury, Safa Kinaneh, Doron Aronson, Offer Amir, Diab Ghanim, Natalia Volinsky, Zaher Azzam, Zaid Abassi

**Affiliations:** ^1^ Department of Physiology, The Rappaport Faculty of Medicine, Technion–Israel Institute of Technology, Haifa, Israel; ^2^ Department of Cardiology, Rambam Health Care Campus, Haifa, Israel; ^3^ Department of Cardiology, B Padeh Medical Center, Poriya, Lower Galilee, Israel; ^4^ Department of Internal Medicine B, Rambam Health Care Campus, Haifa, Israel; ^5^ Department of Laboratory Medicine, Rambam Health Care Campus, Haifa, Israel; ^6^ Faculty of Medicine in the Galilee, Bar-Ilan University, Zefat, Israel

**Keywords:** heart failure, lung, corin, PCSK6, natriuretic peptides

## Abstract

Congestive heart failure (CHF) often leads to progressive cardiac hypertrophy and salt/water retention as evident by peripheral and lung edema. Although the pathogenesis of CHF remains largely unclarified, it is widely accepted that neurohormonal changes and inflammatory processes are profoundly involved in structural and functional deterioration of vital organs including, heart, kidney and lungs. Corin, a cardiac serine protease, is responsible for converting pro-ANP and pro-BNP to biologically active natriuretic peptides (NPs). Although the involvement of corin in cardiac hypertrophy and heart failure was extensively studied, the alterations in corin and PCSK6, a key enzyme in the conversion of procorin to corin, have not been studied in the pulmonary tissue. Thus, this study aims at examining the status of PCSK6/Corin in the lung of rats with CHF induced by the creation of aorto-caval fistula (ACF) between the abdominal aorta and vena cava in SD rats. Rats with ACF were divided into 2 subgroups based on the pattern of their daily sodium excretion, compensated and decompensated CHF. Placement of ACF led to cardiac hypertrophy, pulmonary congestion, and renal dysfunction, which were more severe in the decompensated subgroup, despite remarkable elevation of circulatory ANP and BNP levels. Corin mRNA and immunoreactive peptide were detected in pulmonary tissue of all experimental groups. However, the expression and abundance of pulmonary corin significantly increased in the decompensated animals, but not in the compensated ones. Noteworthy, the expression of PCSK6 and ANP/BNP in the pulmonary tissue followed a similar pattern as corin. The upregulation of pulmonary Corin/PCSK6 and NPs were accompanied by local activation of cathepsin L and certain cytokines including IL-6. In light of the anti-inflammatory role of NPs, we postulate that the obtained upregulation of pulmonary PCSK6/Corin along NPs in rats with decompensated CHF may represent a counterbalance response to the inflammatory milieu characterizing CHF especially in severe cases.

## INTRODUCTION

Heart failure (HF) is endemic in the Western world with continuously rising incidence estimated to affect more than 23 million patients worldwide, especially among aging population [1, 2]. Heart failure is also characterized by high rate of morbidity and mortality and represents one of the leading causes of hospitalization. Only half of the patients survive after 5 years of diagnosis, and the mortality rate reaches 90% in the following 5 years [2–5]. Thus, HF constitutes a substantial clinical and economic burden on both patients and healthcare systems.

Lung edema is the most common complication and leading cause of mortality associated with heart failure [6]. Besides its deleterious impact on symptoms and quality of life, congestion is associated with pulmonary, as well as cardiac, renal, and liver injury, which in turn aggravates clinical outcomes [7, 8]. Therefore, understanding the mechanisms underlying venous congestion and pulmonary edema will allow rapid relief not only of symptoms, but also improvement of prognosis. Activation of the neurohormonal pathways such as renin-angiotensin-aldosterone system (RAAS), sympathetic nervous systems, arginine vasopressin and endothelin plays a fundamental role in the pathophysiology of heart failure in general, and congestion in particular [9, 10]. However, many of the active substances of these hormonal systems such as angiotensin II, aldosterone, catecholamines, and ET-1 possess proinflammatory properties [11, 12]. Likewise, growing evidence suggest that venous congestion plays an adverse stimulatory role in the development of inflammation in CHF [11]. Therefore, besides its cardiac, renal, pulmonary and fluid imbalance manifestations, CHF is considered as inflammatory disease where proinflammatory substances are detected at high concentrations in several vital organs, including heart, kidney, and lung, as well as the circulation [12–16]. These inflammatory processes are profoundly involved in the structural and functional deterioration that occurs in heart failure [11, 12, 15, 16]. While the exact underlying causes of inflammation are not clear, it most likely instigated by intrinsic injury to end target organs, which recruits and activates immune cells of both the innate and adaptive systems.

In heart failure, the natriuretic peptides (NPs) system plays a vital role in opposing the above-mentioned vasoconstrictor/anti-natriuretic neurohormonal system/substances. Atrial natriuretic peptide (ANP) and brain natriuretic peptide (BNP), the main NPs in HF, are secreted mainly from the atria and ventricles, respectively, upon atrial distention and volume/pressure overload [17, 18]. These cardiac prohormones are synthesized as inactive peptides and are activated during their release from the cells. By binding to the NPR-A receptor, ANP and BNP induce the production of cGMP, which in turn promotes vasodilation, diuresis, natriuresis and prevents cardiac remodeling [19]. These protective actions may improve the manifestations seen in HF. Indeed, HF patients exhibit high levels of circulating NPs, serving as biomarkers for HF [20]. Corin is a type-II transmembrane serine protease found mainly in the heart [21, 22]. By converting NPs to their active form [23, 24], corin constitutes an essential player in the regulation of water and salt balance, especially under edematous disease states, including HF. Corin converts ProANP and ProBNP to ANP and BNP with the former being the main substrate. Corin is synthesized as an inactive zymogen composed of an intracellular short domain, a transmembrane domain, and an extracellular region consisting of stem and protease domains [25]. Recently, corin was found to be activated by proprotein convertase subtilisin/kexin-6 (PCSK6), and converted to a two-chain active enzyme through cleavage at Arg-801 [26]. Interestingly, several studies demonstrated that ANP also acts as autocrine/paracrine factor where it modulates various immune functions [27]. There is keen evidence that ANP is locally produced by several immune cells, which also present specific natriuretic receptors. For example, ANP stimulates the phagocytosis of macrophage and killing activity by ROS production, thus improving the innate immunity [28]. Moreover, ANP inhibits lipopolysaccharide (LPS)-induced NO release by macrophages, and promotes the inactivation of Nuclear Factor-kappa B (NF-kB) via cGMP [28]. Zhu *et al.* have demonstrated that ANP reduced the levels of pro-inflammatory cytokines such as IL-1-beta, IL-6, IL-10 and TNF-a in oleic acid-induced acute lung injury in rats [29]. BNP and NT-proBNP levels in ICU patients correlate with inflammatory markers such as CRP and leukocyte count [30]. To the best of our knowledge, pulmonary expression of NPs, their generation machinery and potential involvement in the pathogenesis of lung edema/inflammation in heart failure have not been studied yet.

## RESULTS

### *In vivo* studies

#### Kidney excretory function

**Figure 1:**
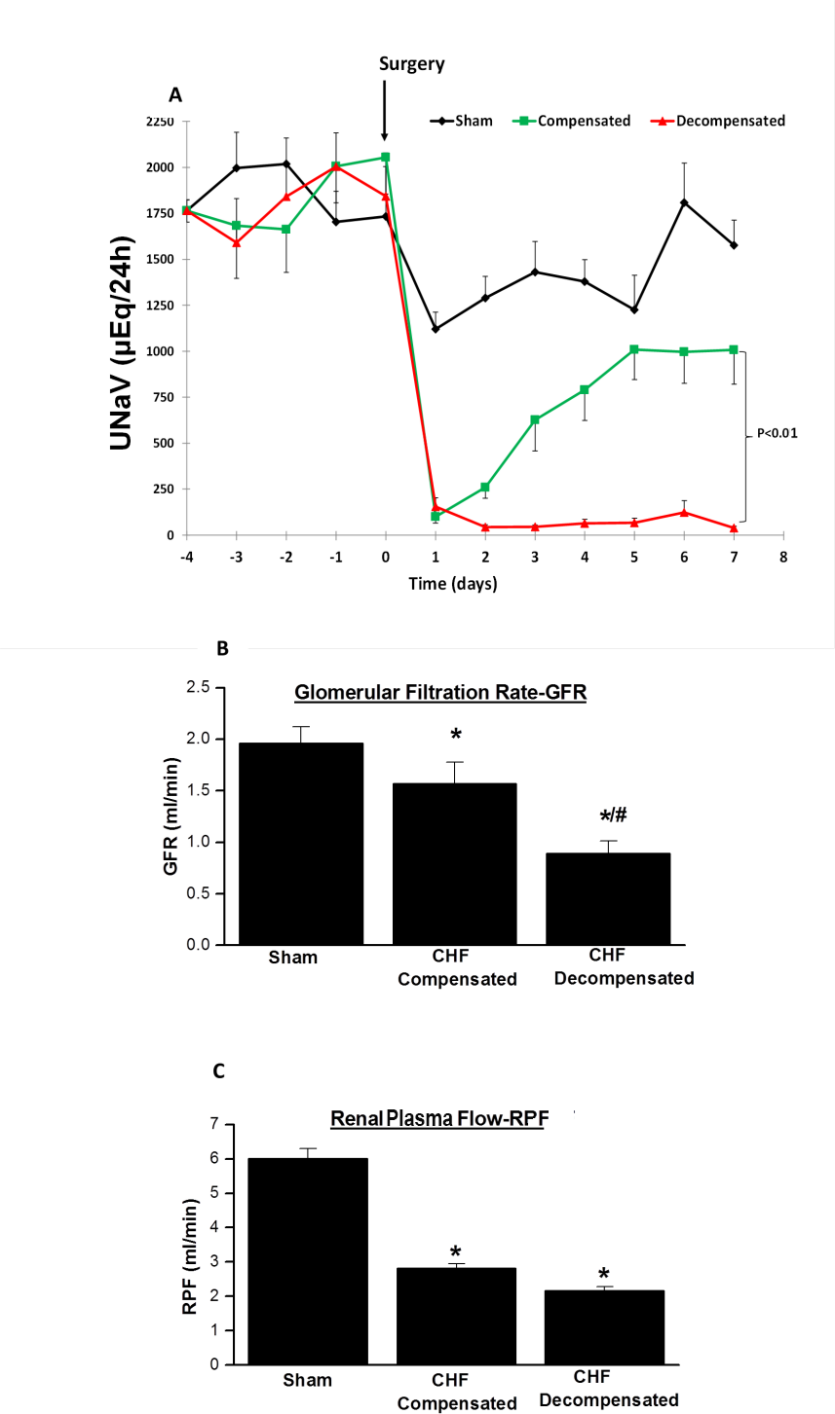
(**A**) Daily urinary sodium excretion (UNaV) in rats with aortocaval fistula (ACF) and in sham controls. Notice two distinct patterns of sodium excretion in rats with ACF, compensated and decompensated rats. The lines representing compensated and decompensated CHF were significantly different (*P <* 0.01, by 2-way ANOVA). Similarly, the lines representing both CHF rats are significantly different from sham- operated animals (*P <* 0.01). (**B**) Glomerular filtration rate (GFR), and (**C**) Renal plasma flow (RPF) in rats with compensated and decompensated CHF and their sham controls. ^*^P < 0.05 vs. sham-operated rats; ^#^*P <* 0.05 vs. compensated rats with ACF. Values are means ± SEM.

Figure 1A depicts the UNaV during baseline and up to 1 week post-operative in sham and rats with compensated and decompensated CHF. Basal daily UNaV ranges between 1,700 and 2000 µEq/24 h. Following surgery, all groups displayed an immediate decline in UNaV. While in sham-operated rats the decrease in UNaV lasted for one day and then returned almost to the presurgical base-line levels, two distinctly different patterns of sodium excretion were evident in rats with ACF. Some of the animals displayed progressive sodium retention, as depicted by the gradual decrease in UNaV through the follow-up period (decompensated CHF). Severe dyspnea and edema, characteristics of CHF, were the main manifestations of this subgroup of animals. The remaining rats with ACF (termed compensated subgroup), displayed increased UNaV in a progressive manner and returned after 1 week to levels comparable to those obtained in the control group.

#### Renal hemodynamics

CHF rats showed impaired kidney function and attenuated renal hemodynamic as compared with sham controls as evident by reduced sodium excretion and lower GFR and RPF (Figure 1B and 1C). Interestingly, kidney dysfunction was in correlation with the severity of heart failure, i.e., decompensated CHF rats exhibited severe reduction in GFR as compared with sham controls and even as compared with the compensated CHF subgroup (Figure 1B and 1C).

#### Heart and lung weights indexes

**Figure 2:**
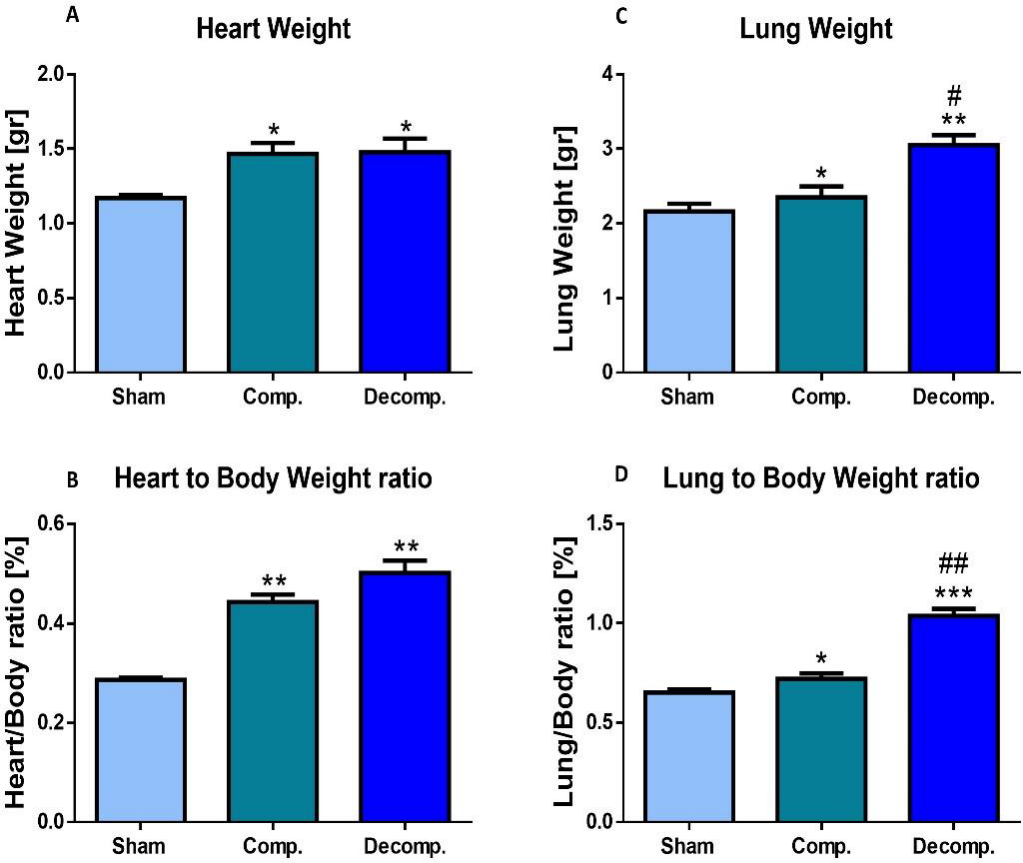
Cardiac and lung weights expressed as absolute values (**A, C**) or as heart weight/body weight ratio (HW/BW%) (**B**), and lung weight/body weight ratio (**D**) of rats with compensated and decompensated CHF. ^*^P < 0.05; ^**^P < 0.01; ^***^P < 0.001 vs. sham-operated rats; ^#^*P* < 0.05; ^##^*P* < 0.01; ^###^*P* < 0.001 vs. compensated rats with ACF. Values are means ± SEM.

The placement of ACF caused an overt increase in the absolute heart weight, due to a marked left and right ventricular hypertrophy and dilation (Figure 2A). Similarly, the heart/body weight, an index of cardiac hypertrophy and heart failure, of rats with compensated and decompensated CHF was significantly elevated relative to the sham rats (0.443 ± 0.015% and 0.50 ± 0.024% vs. 0.28 ± 0.0042, *P <* 0.01), respectively (Figure 2B). The increase in the heart/body weight of the decompensated subgroup was more substantial than that of the compensated animals. This behavior could be attributed to the severity of heart failure or alternatively to the lower body weight gain, characteristic of decompensated CHF rats.

Moreover, absolute lung weight and lung index of the compensated and decompensated CHF rats were higher than those of the sham controls (0.72 ± 0.027% and 1.04 ± 0.036% (*P* < 0.001) vs. 0.65 ± 0.015%, *P* < 0.05), especially in the decompensated subgroup (Figure 2D). Notably, the heart and lung weights of the sham controls were not changed significantly after surgery. These features indicate that this experimental model displays cardiorenal manifestations.

#### Histological analysis of the cardiac and pulmonary tissues

#### Heart

**Figure 3:**
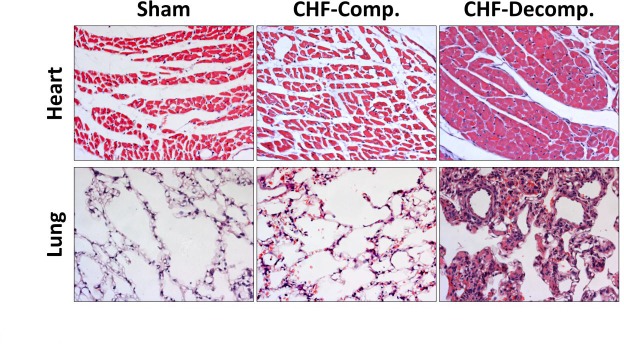
Histological changes in heart and lung tissues from rats with compensated and decompensated CHF and their sham controls Hearts and Lung of the various experimental groups were stained with hematoxylin and eosin. Representative images were obtained at the same magnification (×20).

Heart sections of sham-operated rats and compensated and decompensated CHF subgroups are presented in Figure 3 (Upper panel). The myocytes of the sham-operated group are of normal size (not hypertrophied), the nuclei are located in the periphery and each cell has distinct borders. Overall, the morphology of the cells does not indicate any pathology. Similarly, these characteristics apply to cardiomyocytes from rats with compensated CHF. However, remarkable changes related to severe CHF were detected in heart sections taken from rats with decompensated CHF. As seen in Figure 3, myocytes are hypertrophied, condensed, and enlarged nuclei appear to be closer to the center of the cell with less condensed chromatin, indicating more active cells due to hypertrophy.

#### Lung

Figure 3 (Lower panel) shows histological pulmonary sections of the various experimental groups. Compared with the sham controls, rats with CHF, especially the decompensated subgroup, exhibit simplified and less separated distal airspace. Specifically, rats with decompensated CHF, and to a lesser extent animals with compensated CHF, display more interstitial thickness of the pulmonary tissue along congestion and infiltration of immune cells (probably macrophages) as compared with sham controls.

### Plasma levels of ANP and BNP

**Figure 4:**
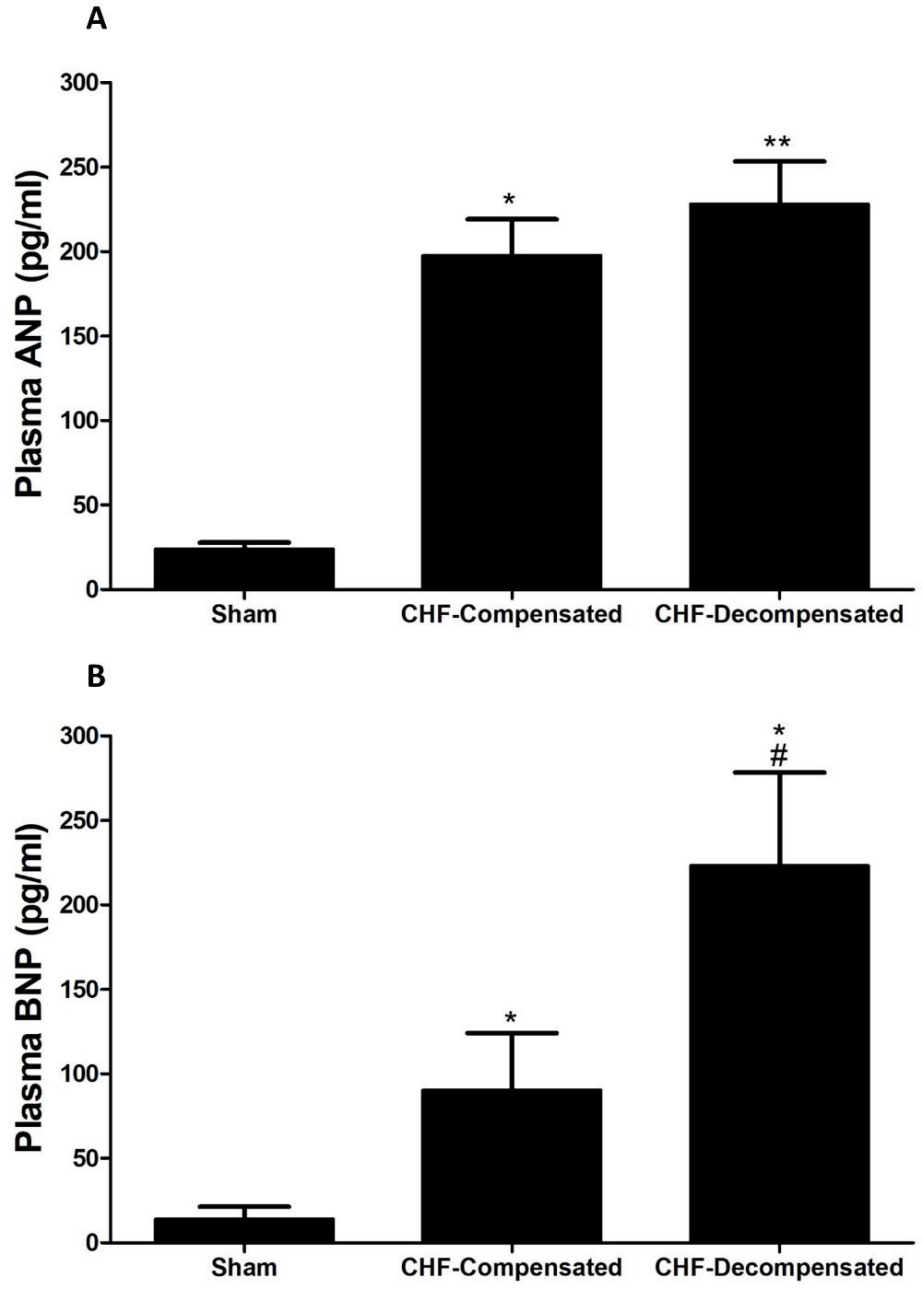
Plasma levels of ANP (**A**) and BNP (**B**) in rats with compensated and decompensated CHF. ^*^P < 0.05 vs. sham-operated rats; ^#^*P <* 0.05 vs. compensated rats with ACF. Values are means ± SEM.

As expected rats with ACF, an experimental model of high-output heart failure, displayed significant elevation in the circulating levels of ANP (∼9-10 fold), which was comparable in compensated and decompensated CHF (Figure 4A). In line with these findings, BNP levels in the blood were increased in rats with ACF as compared with sham controls (Figure 4B). However, this elevation was in correlation with the severity of CHF as was evident by a remarkable and more profound increase in BNP concentrations in the decompensated subgroup (223.0 ± 55.3 pg/ml) as compared with compensated CHF (90.4 ± 33.6 pg/ml).

### Expression of corin-NPs system in the pulmonary tissue

**Figure 5:**
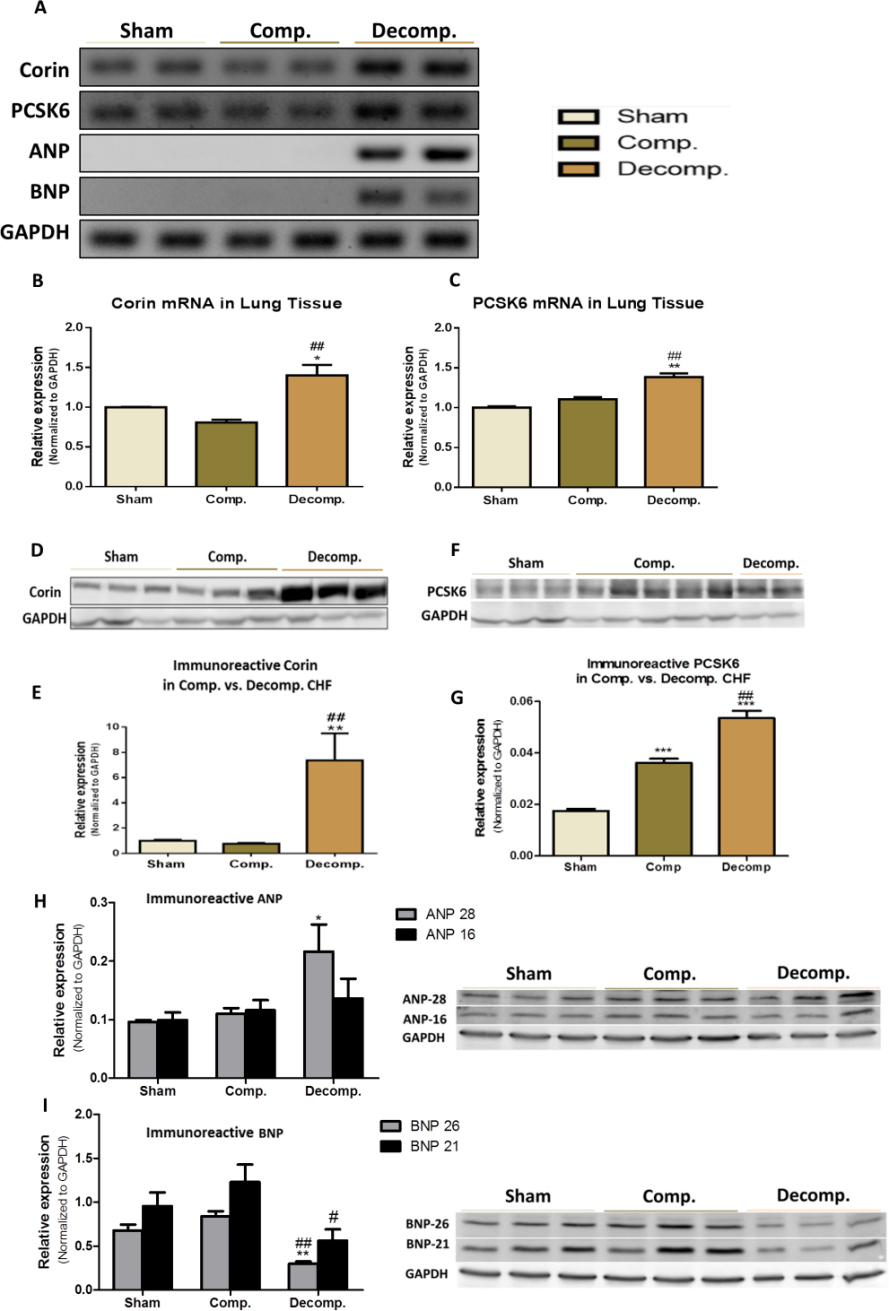
Expression of Corin-NP system in lung tissue of compensated, decompensated CHF and sham controls (**A**) mRNA expression of corin, PCSK6, ANP and BNP. GAPDH expression was used for normalization. Quantification of PCR analysis for corin and PCSK6 mRNAs are depicted in (**B**) and (**C**) panels, respectively. Western blot analysis of the immunoreactive corin (**D**) and its quantification (**E**) in lungs of sham, compensated CHF and decompensated CHF. Western blot of the immunoreactive PCSK6 (**F**) and its quantification (**G**) in lungs of sham, compensated CHF and decompensated CHF. Western blot of the immunoreactive ANP (**H**) and BNP (**I**) in lungs of sham, compensated CHF and decompensated CHF (^*^P < 0.05; ^**^P < 0.01; ^***^P < 0.001). Results are expressed as mean ± SEM. ^*^, represents significant difference of compensated/decompensated CHF vs. sham-operated rats. ^#^, represents significant difference of decompensated CHF group vs. compensated CHF group (^#^*P* < 0.05; ^##^*P* < 0.01; ^###^*P* < 0.001).

In order to study the status of corin and its potential involvement in the pathophysiology of the lungs edema and inflammation in CHF, we first examined whether corin and PCSK6 transcripts are expressed in the lungs of ACF/Sham-operated rats. Noteworthy, by using RT-PCR methodology, we detected substantial amounts of corin and PCSK6 mRNA in the lungs of all experimental groups (Figure 5A). Quantification of corin mRNA expression in compensated CHF rats revealed a slight decrease as compared to sham-operated rats, yet it did not reach statistical significance (0.8076 ± 0.06026 vs. 1.00 ± 0.005919, respectively) (Figure 5B). However, in rats with decompensated CHF, corin mRNA levels were increased compared to compensated CHF (1.40 ± 0.19 vs. 0.81 ± 0.06, respectively; *P <* 0.01) and sham-operated rats (1.40 ± 0.19 vs. 1.00 ± 0.006, respectively; *P <* 0.05). In line with these findings, PCSK6 was also found to be expressed by the lungs in all studied groups. Moreover, PCSK6 mRNA levels were elevated in lungs of decompensated CHF rats, as compared to compensated CHF (1.32 ± 0.08 vs. 1.10 ± 0.046, respectively; *P <* 0.01) and sham-operated animals (1.32 ± 0.08 vs. 1.00 ± 0.0258, *P <* 0.01) (Figure 5C).

After unraveling the presence of Corin/PCSK6 transcripts, we examined the expression and abundance of ANP and BNP hormones in the lungs of the various experimental groups. Interestingly, while lungs of decompensated CHF rats displayed remarkable expression of ANP/BNP mRNA, both sham-operated rats and compensated CHF, did not express these peptides, suggesting local upregulation of the NPs system as CHF is worsening (Figure 5A).

### Immunoreactive levels of corin-NPs system in the pulmonary tissue

Applying western blot analysis supports the pattern of corin expression in the lungs, as was evident by the presence of immunoreactive peptide of this enzyme (Figure 5D) in the pulmonary tissue. Specifically, western blot analysis of the immunoreactive peptide revealed a significant increase in corin abundance in the pulmonary tissue of the decompensated CHF, as compared to compensated CHF (7.37 ± 3.03 vs. 0.76 ± 0.14, respectively; *P <* 0.01) and sham-operated rats (7.37 ± 3.03 vs. 1.00 ± 0.1680, respectively; *P <* 0.01), (Figure 5D and 5E). In agreement with these findings, the abundance PCSK6 (Figure 5F and 5G) was enhanced in rats with CHF, in correlation with disease severity.

Applying western blot analysis for ANP and BNP, revealed the presence of immunoreactive peptides in the pulmonary tissue of the three experimental groups (Figure 5H and 5I). Two bands of ANP corresponding to ∼28 kDa and∼16 kDa (Figure 5H), and of BNP corresponding to ∼26 kDa and 21 kDa were detected (Figure 5I). Most likely, these bands correspond to preproANP/BNP and proANP/BNP. The abundance of ANP of both MWs increased in the lungs of rats with ACF, especially those with decompensated CHF. BNP immunoreactivity was slightly enhanced in rats with compensated CHF, but significantly decreased in the decompensated subgroup.

### Immunofluorescence of corin in the pulmonary tissue

**Figure 6:**
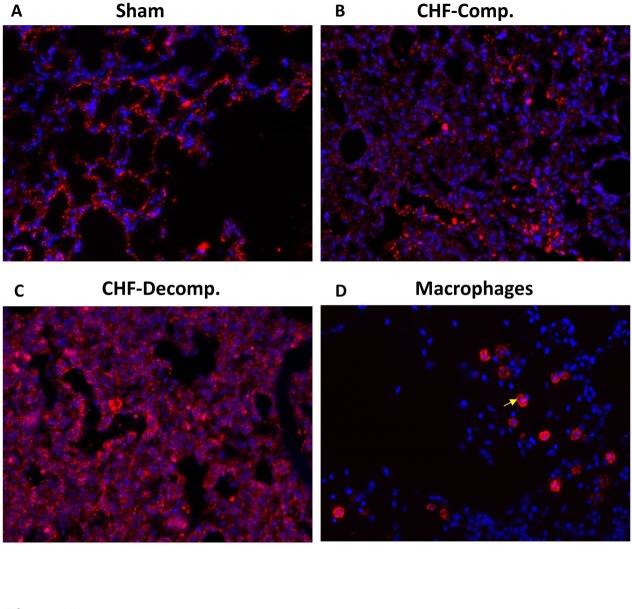
Representative immunofluorescence images of corin immunoreactivity in the pulmonary tissue from the three experimental groups: sham (**A**), compensated (**B**) and decompensated (**C**) CHF. Noteworthy, immunoreactive corin is localized to the pulmonary epithelial cells, as it does not overlap the immunostaining of macrophages (arrow) localized to lung interstitial space of sham controls (**D**).

Representative immunofluorescence images of corin immunoreactivity in the pulmonary tissue from the 3 experimental groups: sham, compensated and decompensated CHF, are presented in Figure 6A–6C. In line with the western blot results, immunofluorescence staining revealed upregulation of corin in the pulmonary tissue of CHF rats, especially the decompensated subgroup (Figure 6A–6C). Noteworthy, immunoreactive corin is localized to the pulmonary epithelial cells (Figure 6A–6C). Specific immunostaining of macrophages revealed different localization and immunofluorescence patterns (Figure 6D), which does not overlap the corin staining. Specifically, inflammatory cell infiltration was mostly observed in the interstitium, indicating that corin expression is specific to epithelial cells, rather than the immune system.

### Na^+^, K^+^ ATPase and ENaC abundance in the pulmonary tissue

**Figure 7:**
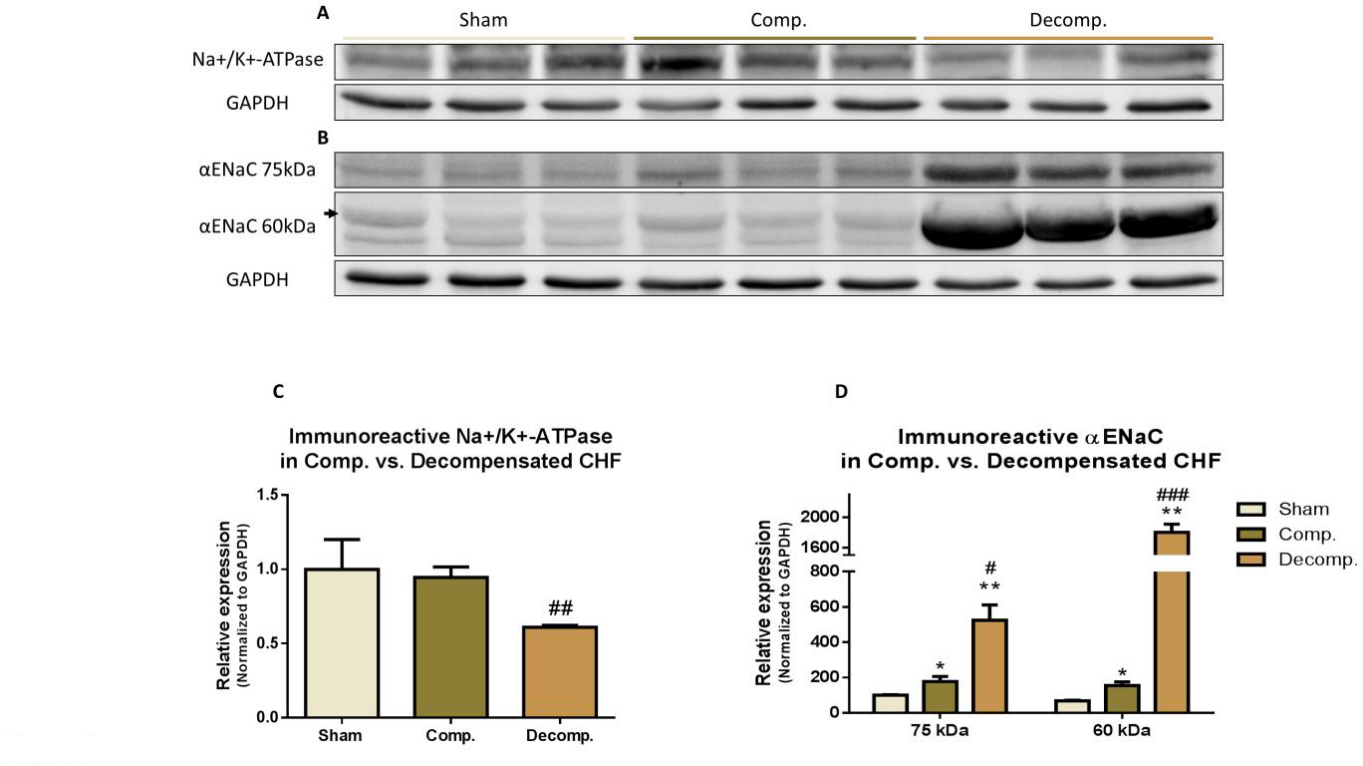
(**A**) Representative abundance (western blot) of Na^+^/K^+^ ATPase (alpha 1 subunit) in lung tissue of compensated and decompensated CHF rats and their sham controls; (**C**) Quantification Na^+^/K^+^ ATpase (alpha 1) abundance. (**B**) Representative abundance (western blot) of αENaC in lung tissue of compensated and decompensated CHF rats and their sham controls; (C) Quantification Na^+^/K^+^ ATPase (alpha 1) abundance. (**D**) Quantification αENaC abundance. Results are expressed as mean ± SEM. ^*^represents a significant difference between CHF subgroups and sham controls (^*^P < 0.05; ^**^P < 0.01; ^***^P < 0.001). ^#^represents significant difference of decompensated CHF group vs. compensated CHF group (^#^*P* < 0.05; ^##^*P* < 0.01; ^###^*P* < 0.001). The bars represent mean ± SEM.

Figure 7 depicts the abundance of the αNa+, K+ATPase and αENaC in the various experimental groups. We determined the immunoreactive protein levels of both αNa+, K+ATPase and αENaC in compensated and decompensated CHF as compared with sham controls. A significant decrease in αNa+, K+ATPase was observed in the decompensated subgroup, but not compensated one (Figure 7A). Western blotting of αENaC showed higher levels of ∼75-kDa ENaC in lung homogenates of decompensated CHF rats, but not of compensated CHF animals compared with sham controls (Figure 7B). Similarly, the levels of the lower band of ENaC (∼60-kDa) were significantly higher in decompensated group as compared with compensated CHF and sham controls (Figure 7D).

### Cytokines and Cathepsin-L abundance in the pulmonary tissue

**Figure 8:**
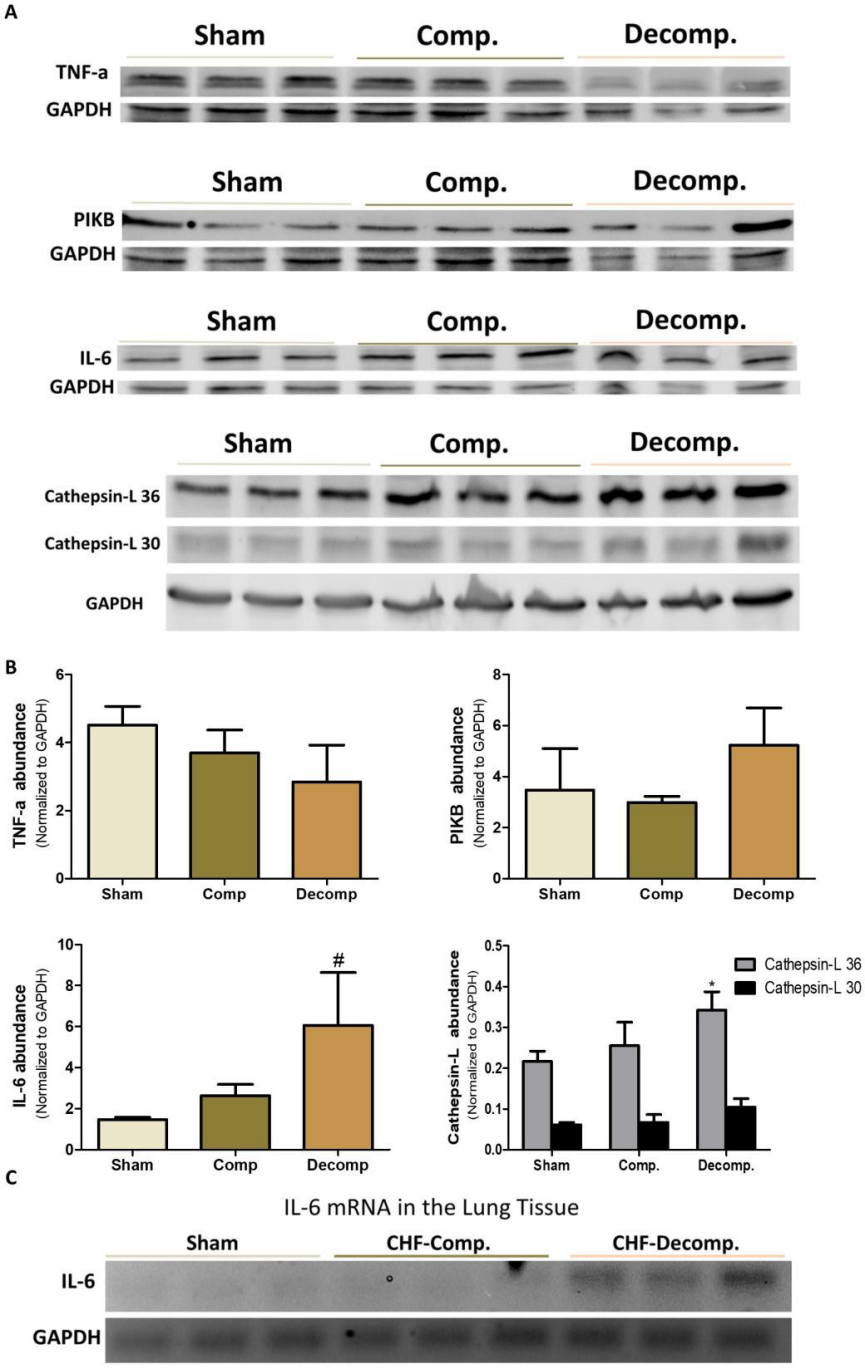
(**A**) Immunoreactive levels of IL-6, TNFα, IκB and Cathepsin L in lung tissue of compensated and decompensated CHF and their sham controls. (**B**) quantification of the Western blot results. (**C**) mRNA expression of IL-6 in lung tissue of compensated, decompensated CHF and sham controls. ^*^, Represents significant difference of compensated/decompensated CHF vs. sham-operated rats. ^#^, Represents significant difference of decompensated CHF group vs. compensated CHF group.

In addition to investigating the status of the corin/NPs system, we examined the alteration in TNFα, IL-6, IκB, and Cathepsin-L abundance in the lungs of rats with compensated and decompensated CHF as compared with sham controls. As shown in Figure 8A, 8B we found that rats with decompensated CHF displayed higher pulmonary immunoreactive levels of IL-6 and Cathepsin-L, a lysosomal enzyme associated with inflammatory diseases, as compared with sham-operated rats and compensated CHF. Likewise, applying RT-PCR revealed expression of IL-6 only in the subgroup of animals with decompensated CHF (Figure 8C).

Interestingly, expression of TNFα decreased in rats with ACF, especially those with decompensated CHF (Figure 8A, 8B). In line with these findings, IκB, a family member of cellular proteins that function to inhibit NF-κB, slightly increased in the decompensated CHF subgroup.

## DISCUSSION

The present study provides novel information concerning the status of corin and PCSK6 in the pulmonary tissue of rats with CHF. As expected, CHF rats exhibited typical pulmonary and cardiac changes characterizing CHF, including impaired kidney function and renal hypoperfusion and cardiac hypertrophy. Regarding the latter, placement of ACF caused an overt increase in heart/body weight, an index of cardiac hypertrophy and heart failure, one week after surgery. Likewise, lung weights of CHF rats were higher than those of sham controls. These deleterious changes were more profound in the decompensated subgroup, supporting the reliability of ACF as experimental model of CHF to study the mechanisms underlying the pathogenesis of clinical heart failure, which similarly displays two distinct patterns, namely compensated and decompensated settings. Although the involvement of corin in cardiac hypertrophy and heart failure was extensively studied, to the best of our knowledge this is the first study to examine the alterations in corin and PCSK6 in the pulmonary tissue during CHF. Specifically, corin mRNA and immunoreactive peptide were detected in pulmonary tissue of all experimental groups, however, the expression and abundance of this enzyme significantly increased in the decompensated, but not compensated animals. Moreover, expression of PCSK6 and ANP/BNP in the pulmonary tissue followed a similar pattern as corin. The upregulation of pulmonary corin/PCSK6 and NPs was accompanied by local activation of Cathepsin L and pro-inflammatory cytokines including IL-6, suggesting a potential correlation between the PCSK6/Corin/NPs axis and the inflammatory milieu characterizing CHF, particularly in the decompensated stage.

So far, several studies have examined the alterations of corin in the myocardium and addressed its involvement in the pathogenesis of heart failure [31–33]. In this context, patients with decompensated heart failure have lower levels of circulating corin along decreased cleavage of the pro-atrial natriuretic peptide [31]. Similarly, corin decreased in the atrium 4 weeks after the induction of experimental heart failure by an aortocaval shunt [32]. In contrast, overexpression of corin mRNA was observed in the noninfarcted LV myocardium 8 weeks after the induction of hypertrophied ventricle induced by LAD ligation in rats [33]. Unfortunately, none of these studies examined the expression of this key enzyme in the pulmonary tissue. Thus, the detection of corin transcripts and immunoreactive peptide in the lungs on one hand, and its upregulation in decompensated CHF on the other may provide new insights into the pathogenesis of lung edema and the inflammatory aspect of heart failure. This notion is further supported by the fact that corin deficiency in mice was associated with reduced sodium excretion as well as salt- sensitive hypertension along cardiac hypertrophy [34, 35]. Likewise, PCSK6 deficient mice display salt-sensitive hypertension [26]. In agreement with the beneficial role of corin, overexpression of this enzyme in mice with dilated cardiomyopathy enhanced heart contractile function, as was evident by improved fractional shortening and ejection fraction, and significantly reduced heart failure as assessed by lung water and alveolar congestion [36]. Taken together, our observation that rats with decompensated CHF displayed remarkable upregulation of corin, PCSK6 and NPs suggests that this axis may play a counterregulatory role in the development of lung edema. Interestingly, despite the upregulation of the enzymatic machinery of ANP and BNP generation, the immunoreactive levels of the latter were substantially lower in the lung of decompensated CHF animals. BNP and ANP play an important physiological role in the lungs, where they affect salt and water clearance and vascular permeability [37, 38]. Therefore, the impaired generation of BNP in parallel to ANP abundance in the lungs may contribute to the development of congestion in rats with severe heart failure, although a direct cause and effect relationship needs to be established.

The role of the NPs in modulating the immune system in cardiovascular diseases, including heart failure, is continuously emerging [27, 39]. In this context, it was found that ANP is locally produced by several immune cells, which also present specific receptors to this natriuretic hormone [27]. Activation of these binding sites by ANP stimulates the macrophage as assessed by enhanced phagocytosis and the killing activity via ROS overproduction [28]. Moreover, ANP enhances the inactivation of Nuclear Factor-kappa B (NF-kB) via cGMP [28]. Zhu *et al.* have demonstrated that ANP reduced the levels of pro-inflammatory cytokines such as IL-1beta, IL-6, IL-10 and TNF-a in oleic acid-induced acute lung injury in rats [29]. At the clinical level, BNP and NT-proBNP levels in severely ill patients correlate with inflammatory markers such as CRP and leukocyte count [30]. Our findings that both ANP and BNP are overexpressed in the pulmonary system along with IL-6 and Cathepsin L in rats with decompensated CHF, hint possible interaction between NPs and certain cytokines in the lungs. Support for this concept is derived from the observation that patients with acute decompensated heart failure (ADHF) exhibit higher levels of C-reactive protein (CRP) and IL-6 compared with healthy controls [40]. The interaction between NPs and cytokines was demonstrated by the administration of ANP to rats with acute kidney injury induced by I/R where it inhibited mRNA expression of TNF-α, IL-1β, and IL-6 in the lung and kidney. ANP also attenuated the histological localization of TNF-α, IL-6, and NFκB in the renal and pulmonary tissues. Likewise, it attenuated the increase in the plasma concentrations of pro-inflammatory cytokines including IL-1β and IL-6 induced by renal failure [41]. Our findings that rats with decompensated CHF display lower levels of immunoreactive TNF-α as compared with sham-operated rats and even compensated CHF animals is in contrary to the clinical findings where TNF-α levels are increased in heart failure [11]. These differences could be attributed to the etiology and duration of heart failure and the presence of comorbidities including infections.

**Figure 9:**
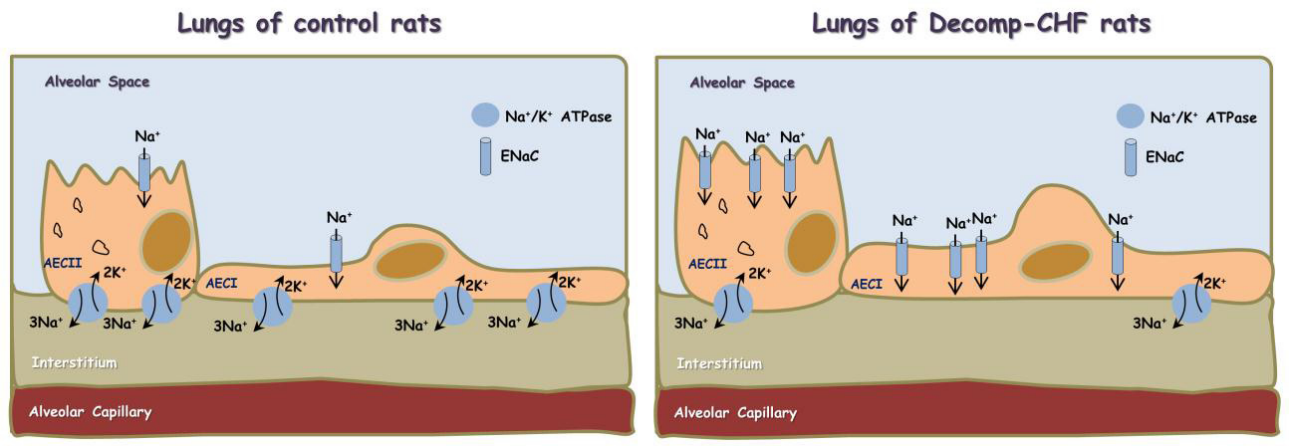
Scheme showing alveolar fluid clearance process in the lung epithelium under normal condition and during decompensated CHF Sodium is actively transported from alveolar space to the lungs’ interstitium and blood vessels; achieved mainly by apical ENaC and basolateral Na^+^/K^+^ ATPase located to Alveolar Epithelial Cells type I (AECI) and ATII (AECII). This results in the formation of osmotic gradient, which drives transcellular and paracellular movement of water molecules. In decompensated CHF there is upregulation of ENaC in the apical side and downregulation of Na^+^/K^+^ ATPase in the basolateral side, which may contribute to the development of lung edema.

Finally, our findings that local generation of NPs correlates with increased ENaC expression is of special interest, since the obtained upregulation of ENaC might explain the development of lung edema in decmpensated CHF on the one hand, and the regulation of ENaC by NPs on the other. Specifically, active Na^+^ transport across the alveolar-capillary barrier is important in keeping the airspaces free of fluid in healthy conditions and for the resorption of lung edema in pathologic conditions. In this context, Na+ enters the alveolar epithelial cells through apical amiloride-sensitive ENaC, and is actively pumped out of the cell by the Na,K-ATPase located in the basolateral membrane [42–46] (Figure 9). It has been shown that NPs regulate ENaC activity in the kidney via the second messenger guanosine 3′, 5′-cyclic monophosphate (cGMP) [47]. At the pulmonary level, it was shown that in the presence of left atrial hypertension, ANP inhibits the stimulatory effect of β-adrenergic agonists on alveolar fluid clearance [48]. In addition, it has also been shown that ANP post-treatment ameliorated injuries in kidney and lung by direct tissue protective effect and anti-inflammatory effects, which potentially inhibited inter-organ crosstalk [49]. Based on the current data, we postulate that upregulation of ENaC in the apical membrane along reduced expression of Na^+^, K^+^ ATPase in the basolateral membrane contributes to the development of lung edema mainly in the decompensated subgroup (Figure 9). The alterations in NPs production by the pulmonary tissue suggest that interplay between this system and ENaC and Na^+^, K^+^ ATPase as well as the inflammatory response may exist under severe CHF. However, additional studies are needed to test whether ENac changes result from either lung edema or changes in NPs, or interactions between the two. In short, it would be of interest to learn more about the interactions between lung edema, NPs and ENac, and to better understand how these interactions relate to lung physiology. These interactions might be studied more mechanistically in an epithelial cell line, where it might be possible to either up- or down-regulate NP production.

In summary, the current study demonstrates that pulmonary system expresses NPs and its generation machinery. In addition, the upregulation of PCSK6 and corin in rats with decompensated CHF along lung congestion and activation of key cytokines suggest a potential involvement of these systems in the pathogenesis of lung edema/inflammation characterizing severe CHF. These findings may serve as a basis for improvements in pharmacological therapy of CHF, which remains largely unchanged over the past 20 years. Yet, additional studies are still required in order to establish possible interrelationships between lung edema, ENaC upregulation and altered local NPs production.

## MATERIALS AND METHODS

Studies were performed on male Sprague-Dawley rats (Harlan Laboratories, Jerusalem, Israel), weighing 300-350 g. The rats were housed in individual metabolic cages in a temperature-controlled room and fed a standard rodent diet and tap water *ad libitum*. Daily urinary volume and urinary sodium concentration were measured throughout the entire study period (beginning 5 days prior to surgery). Experiments were performed according to the “Guide for the Care and Use of Laboratory Animals” (NIH Publication No. 85-23, revised 1996) as approved by the local institutional committee for supervision of animal experiments.

### Experimental model

An aorto-caval fistula (ACF) was surgically created between the abdominal aorta and the inferior vena cava, as reported in detail in our laboratory previous studies [10, 50–52]. Briefly, under sodium pentobarbitone anesthesia (70 mg/kg BW, i.p.), the abdominal aorta and inferior vena-cava were exposed through a mid-abdominal incision. The outer wall of the vena-cava was opened and a fistula (1.2 mm outer diameter) was surgically created in the common wall between the vessels. The opening in the wall of the vena cava was closed with a continuous suture. A matched group of sham-operated rats that underwent laparotomy only served as control. The animals were allowed to recover and then returned to their metabolic cages, for monitoring of urine output and sodium excretion for additional 7 days. Based on their urinary sodium excretion (UNaV), rats with ACF were divided into 2 subgroups: compensated (UNaV > 1200 μEq/day) and decompensated (UNaV < 200 μEq/day). The various experimental groups were subjected to *in vivo* or *in vitro* protocols.

### *In vivo* protocols

#### Clearance studies

Three groups of rats were studied: 1) sham operated controls (*n =* 8), 2) rats with compensated CHF (*n =* 8), and 3) rats with decompensated CHF (*n =* 7). On the day of the experiment, animals from the various groups were anesthetized with Inactin (100 mg/kg, BW i.p.) and prepared for clearance studies. Briefly, after tracheotomy, polyethylene tubes (PE_50_) were inserted into the carotid artery, jugular vein, and urinary bladder for blood pressure monitoring, infusion of solutions, and urine collection, respectively. A solution of 2% inulin and 0.5% p-aminohippurate (PAH) in 0.9% saline was continuously infused at a rate of 1.0–1.5% of BW/h throughout the experiment. Mean arterial pressure (MAP) was continuously monitored with a pressure transducer (model 1050.1, UFI, Morro Bay, CA, USA) connected to the carotid arterial line. After a 60 min. equilibration period, two baseline 30 min clearance periods were obtained. Blood samples were obtained at the end of each clearance period. The values from the two baseline collection periods were averaged and combined in all experimental groups. At the end of the experiments, animals were sacrificed and the hearts and lungs removed and weighed. Cardiac/body weight ratio (HW/BW%), an index for cardiac hypertrophy, was calculated in the various groups of animals. Likewise, the lung weight to body weight ratio (LW/BW%) was calculated.

### *In vitro* protocols

### *Heart* and lungs fixation

Additional groups of rats with CHF as well as sham controls (*n =* 3) were anesthetized and their lungs were fixed via right ventricle perfusion, first with 120 ml phosphate buffered saline (0.01 M PBS, pH 7.4) containing heparin (5 U/ml), then with 220 ml of ice-cold 4% paraformaldehyde in 0.01 M PBS, pH 7.4 containing sucrose 4%. Heart and Lungs from the different experimental groups were removed and embedded in 0.01 M PBS, pH 7.4 containing sucrose 4% and paraformaldehyde 4%. Heart and lungs tissues were then progressively dehydrated in graduated alcohol concentrations (70–100%) and embedded in paraffin. For general histomorphology, 5 μm sections were stained with hematoxylin and eosin (H&E).

### Immunofluorescence

Five μm-thick paraffin sections of the pulmonary tissue were deparaffinized and rehydrated. Then slides were subjected to antigen retrieval by boiling (10 min) in 10 mM citrate buffer, pH 6.0. Slides were then incubated with 5% normal donkey serum (NDS) in phosphate buffered saline (PBS) containing 0.3% Tween-20 for 60 min to block nonspecific binding and incubated overnight at 4° C with antibodies directed against corin (1:100, Ab125254) or F4/80 (1:100, ab100790; Abcam, Cambridge, UK) antibodies diluted in blocking solution. Cy™3 Donkey Anti-Rabbit IgG was used as secondary antibody (Jackson Laboratories PA, USA) together with DAPI Fluoromount-G^®^ for nuclear staining. Images were captured using a Zeiss LSM 700 Confocal microscope and analyzed with Zen software (Carl Zeiss).

### Western blot analyses

Lungs tissue samples were homogenized on ice and centrifuged at 4° C for 5 min at 3000 RPM. The homogenized tissue was then lysed in RIPA buffer (150 mM NaCl, 1% NP40, 50 mM Tris pH 8.0, 0.5% sodium deoxycholate and 0.1% SDS) supplemented with a cocktail of protease inhibitors (Roche) in rotation at 4° C for 20 min, and then centrifuged at 4° C for 10 min at 12,000 RPM. The cleared supernatant was collected and protein concentration was determined (Bradford reagent, Sigma). Equal amounts of extracted proteins (40–60 μg) were loaded and run on a 7.5% SDS–polyacrylamide gel and were transferred to nitrocellulose membrane. The membrane was incubated in blocking buffer, TBS-T (Tris-buffered saline, 0.1% Tween 20) containing 5% (w/v) BSA, and probed with the appropriate primary antibodies: anti-corin (1:1000, Ab125254), anti-GAPDH (1:1000, sc-25778), anti-ANP (1:200 sc-18811, Santa Cruz); anti-BNP 1:200, sc-18818); goat polyclonal Cathepsin L 1:1000, sc-6498, Santa Cruz), anti-IL-6 1:300, sc-1266; anti-TNF-a (1:1000, ab66579, Abcam); anti PIkB (1:1000, # 2859, cell signalling); alpha 1 subunit of Na+,K+ ATPase (1:250, ab7671, Abcam), and anti-alpha subunit of ENaC (Alomone ASC-030, Jerusalem, Israel). After washing with TBS-T, the immunoreactive proteins were visualized with horseradish-conjugated goat anti-rabbit secondary antibody (1:25,000 Jackson, 111-035-144) and chemiluminescent substrate.

### Gene expression analysis by RT-PCR

Total RNA was isolated from snap-frozen tissue samples using TRIzol^®^ Reagent (Life Technologies), according to the manufacturer’s instructions, and quantified by spectrophotometry using NanoDrop 2000. After oligo (dT)-primed reverse transcription of 1000 ng total RNA, the resulting single-stranded cDNA was used for PCR. PCR conditions were as follows: an initial denaturation step at 95° C for 3 minutes, 30 cycles of denaturation at 95° C for 30 seconds, and hybridization at 60° C for 30 seconds followed by elongation at 72° C for 1 minute. Finally, the PCR reaction was terminated by incubation at 72° C for 5 minutes. GAPDH was used as an internal standard. The following primers were used:

Corin: F(5′-GAAGACTGTAAGGACGGGAGTGA-3′) R(5′-GTCAAGGCAACC-CCGATCT-3′), GAPDH: F(5′-GTGCCAGCCTCGTCTCATAG-3′), R(5′-GAGAAGGCAGCCCTGGTAAC-3′); PCSK6: F(5′-GCTCACGGCTACCTCAACTT-3′), R(5′-CTGTCTCTTGACCCTGCGTT-3′); NPPA: F(5′-CCTGGACTGGGGAAGTCAAC-3′) R(5′-GCAGCTCCAGGAGGGTATTC-3′), NPPB: F(5′-TCCTTAATCTGTCGCCGCTG-3′), R(5′-CGCCGATCCGGTCTA-TCTTC-3′); IL-6, F (5′-CGAGCCCACCAGGAACGAAAGTC-3′), R (5′-CTGGCT-GGAAGTCTCTTGCGGAG-3′).

### Chemical analyses

Urine volume was determined gravimetrically. The concentrations of sodium in plasma and urine were determined by a flame photometer (model IL 943, Instrumentation Laboratory, Italy). Concentrations of inulin and PAH in plasma and urine were measured by colorimetric methods. Renal plasma flow (RPF) and glomerular filtration rate (GFR) were estimated as the infusion clearances of PAH (CPAH = UPAH*V/PPAH) and inulin (Cin *=* Uin*V/Pin), respectively. Plasma ANP was determined using an ELISA (Atrial Natriuretic Factor 1-28 EIA Kit, Peninsula Laboratories, San Carlos, CA, USA) following an extraction procedure of plasma samples according to the manufacturer’s protocol for C18 Sep-column extraction method (C18 Sep-columns and Extraction Kit, Peninsula Laboratories). Plasma BNP was determined by ELISA (AssayMax rBNP-32 ELISA kit, Assaypro, St. Charles, MO, USA).

### Statistical analysis

Data are presented as mean ± SEM. Comparison between two parametric groups was performed using the unpaired Student *t* test after testing for equality of variances. When more than two paired groups were tested the one-way analysis of variance (ANOVA) test for repeated measurements, followed by the Bonferroni post-hoc test for multiple comparisons was applied.

## Abbreviations

ACF: Aorto-caval fistula
ADHF: Acute decompensated heart failure
ANP: Atrial natriuretic peptide
BNP: Brain natriuretic peptide
cGMP: Cyclic guanosine monophosphate
CHF: Congestive heart failure
ENaC: Epithelial sodium channel
ET-1: Endothelin
HF: Heart Failure
LPS: lipopolysaccharide
IL-6: Interleukin 6
IL-10: Interleukin 6
I/R: Ischemia reperfusion
LV: Left ventricle
MAP: Mean arterial pressure
NF-kB: Nuclear Factor-kappa B
NPs: Natriuretic peptides
PCSK6: Proprotein Convertase Subtilisin/Kexin Type 6
PAH: p-aminohippurate
RAAS: Renin-angiotensin-aldosterone system
ROS: Radical oxygen species
TNFα: Tumor necrosis factor α
UNaV: Urinary sodium excretion
